# Exploring the Perception and Awareness of Dental Students and Interns in Managing and Treating Pregnant Patients

**DOI:** 10.7759/cureus.52567

**Published:** 2024-01-19

**Authors:** Mahir A Mirah, Amnah A Algarni, Rasha S Alafaleg, Jameel A Abuljadayel, Shihanah Alotaibi, Abdulmajeed Baik, Yara A Alnazzawi, Rasha O Aldadjan, Arwa Bafail

**Affiliations:** 1 Department of Restorative Dental Sciences, College of Dentistry, Taibah University, Madinah, SAU; 2 Department of Dental Education, College of Dentistry, Qassim University, Qassim, SAU; 3 Department of Preventive Dentistry, Faculty of Dental Medicine, Umm Al-Qura University, Makkah, SAU; 4 College of Dentistry, Taibah University, Madinah, SAU

**Keywords:** practice, attitude, knowledge, interns, dental students, confidence levels, pregnant women

## Abstract

Background

Undergraduate dental students and interns express reluctance to deal with pregnant women in the dental office due to the fear of medical consequences on the fetus. This study aims to explore the knowledge, attitudes, and practices of dental students and interns regarding the dental considerations of pregnant women in three dental schools in Saudi Arabia.

Methodology

This cross-sectional study was performed between October and December 2023 and targeted the clinical academic years of dental students and interns at three dental schools in Saudi Arabia, namely, Taibah University, Umm Al-Qura University, and Qassim University. Using the convenience sampling method, 223 students were invited to respond to a questionnaire constructed from previously published studies and reviewed by experts in the field. It consisted of 15 questions divided into four sections. Descriptive analysis and chi-square test were conducted to assess the difference between the different sociodemographic groups and students’ educational levels with the significance level set at p-values <0.05.

Results

Overall, 223 complete responses were received, with a response rate of 97%. Demographics included 99 (44.40%) males and 124 (55.60%) females, with a reasonable distribution across academic years. Knowledge assessment findings revealed that 114 (51%) participants acquired adequate knowledge. Regarding participants’ attitudes, 112 (50%) found the curriculum-centered information sufficient. In addition, 173 (77.6%) participants chose lectures/books as their preferred source of information. Almost 138 (61.9%) participants did not feel confident in managing pregnant women. The chi-square test revealed that educational level significantly impacts the knowledge and practice of the participants, regardless of their gender (p < 0.05).

Conclusions

Both students and dental interns showed adequate-to-good levels of knowledge and attitude. Both need more exposure to clinical situations with pregnant women during their undergraduate dental training programs to boost their confidence level and enhance their readiness to practice dental procedures with such a group of patients after graduation.

## Introduction

Numerous anatomical, physiological, and hormonal changes during pregnancy make pregnancy a unique time in a woman’s life. These changes can have an indirect impact on oral health [1]. Women’s physical and mental well-being is challenged during pregnancy, in addition to hormonal shifts and changes in their oral flora which makes them at risk of poor dental health [2].

Many dental health problems are correlated with pregnancy, such as caries, gingival inflammation, and mouth dryness [3]. Pregnancy is associated with changes in the oral cavity, including changes in soft and hard tissues [3]. Carbohydrate loading is associated with increased dental decay when snacking frequency increases [4]. Erosive tooth wear of the lingual surfaces of the teeth is noticed in pregnant women; it is caused by exposure to stomach acids, which can occur by morning sickness and vomiting [5,6]. Gingivitis symptoms, including bleeding, redness, swelling, and pain, appear in the second trimester and peak during the eighth month of pregnancy [7]. Untreated gingivitis during pregnancy has been linked to premature birth and low birth weight [8]. Granuloma gravidarum, commonly appearing during pregnancy, can be precipitated by bacteria, calculus, sharp parts of fractured teeth, or food impaction [9].

Most women decide not to receive dental treatment during pregnancy, which is not an excuse. This may be due to the obstetrician’s lack of interest, the patient’s lack of communication, or the dentist’s uncertainty about the safety measures during care due to misinformation or a lack of knowledge. Delaying the required dental care may have unexpected consequences for the mother and fetus. Surprisingly, half of the pregnant women with dental issues visit the dentist. Dental professionals and students are responsible for educating women about the need and importance of getting dental treatment when needed, even when pregnant [10]. Patients are typically hesitant to undergo dental radiography because of worry and anxiety about the potential for cancer or genetic anomalies, which, in turn, may delay critical treatment and severely affect the health of both the mother and fetus [11].

The Food and Drug Administration (FDA) has recommended a risk-based categorization system for pharmaceuticals (A, B, C, D, and X). Drugs in categories A and B are considered safe for humans. Categories A and B differ in whether or not the medicine has been tested on humans. Category C drugs are those for which teratogenic risks cannot be eliminated. Drugs in category D had positive evidence of fetus danger. Finally, drugs in category X should not be prescribed under any circumstances [12]. Local anesthetics, such as lidocaine and prilocaine, have not been proven to be teratogenic in human or animal studies, in contrast to bupivacaine, articaine, and mepivacaine, which have shown some teratogenic risk [13].

According to a survey conducted in Australia, most dentists are aware of the procedures and basic dental care that are safe to perform during pregnancy. On the other hand, some previous reports have suggested that dental undergraduates showed a lack of knowledge regarding some aspects of pregnant patients’ dental care, such as proper patient posture during dental treatment [2,14].

The literature shows scarce information about the knowledge level of undergraduate dental students concerning dental considerations of pregnant women in Al Madinah, Makkah, and Qassim dental schools in Saudi Arabia. This study aimed to explore the level of awareness and knowledge of dental interns and students in managing and treating pregnant women at Taibah University, Umm Al-Qura University, and Qassim University.

## Materials and methods

This cross-sectional study was conducted between October and December 2023. A self-structured questionnaire was used among dental students and interns at Taibah University, Al Madinah, Umm Al-Qura University, Makkah, and Qassim University, Buraydah. According to the predetermined inclusion criteria, students from the fourth, fifth, and sixth year and dental interns at the previously mentioned dental schools were included. Students who were unwilling to participate or below the fourth level were excluded.

The questionnaire consisted of multiple-choice questions constructed and edited by experts from the previously mentioned dental schools after reviewing similar publications [1,8]. The survey included 15 questions, divided into three sections to assess participants’ knowledge, attitude, and practice, in addition to a demographic data section. The contents of the survey were tested on a random pilot sample of 15 participants (five from each school), which were not included in the study, and performed to confirm the clarity of the survey questions. Some changes in the wording were made accordingly. The questionnaire covered the main areas essential to providing safe and effective dental care for pregnant patients. For example, the common oral diseases occurring during pregnancy, periodontitis as a risk factor for pregnancy complications, and the best time and position to provide dental care for pregnant women. In addition, questions regarding the safest medications and anesthesia for pregnant patients were also covered, as well as the safety of dental radiograph exposure during pregnancy. The survey used in this study is provided in the Appendices.

The sample size calculations were done using the convenience sampling method utilizing the Raosoft website. The settings were adjusted to determine the sample size with a 5% margin of error, 99% confidence level, 50% response distribution, and an estimated 300 students from the three dental schools following the inclusion criteria. Using these parameters, the calculated sample size was 207. This number was amplified by around 10% to compensate for the expected unwillingness to participate among the invited sample. Consequently, all students who fulfilled the inclusion criteria were digitally invited and followed up during the data collection period, aiming for 228 participants.

To assess knowledge, attitude, and practice, the correct or best answer was coded 1, and the rest were coded 0. The total score of each participant’s answers was divided by the number of questions multiplied by 100 to obtain the percentage value. The percentage values that ranged between 66.6% and 100% were considered a good level of achievement. Values between 33.3% and 66.6% were considered adequate levels, and values between 0% and 33.3% were considered poor achievement.

The research protocol received ethical approval from the ethics committee of the College of Dentistry, Taibah University (protocol number: TUCDREC/041023/MAMirah).

Data were transferred from Google Sheets into the SPSS version 20 (IBM Corp., Armonk, NY, USA) for analysis. Descriptive analysis included frequencies and percentages. The chi-square test was conducted to assess the difference between the different sociodemographic groups and students’ educational levels at a significance level of p < 0.05.

## Results

Of the calculated and aimed sample size, 223 participants completed the questionnaire, with a response rate of 97%. Among the participants, 99 (44.40%) were males, and 124 (55.60%) were females. Most participants were from Taibah University (113, 50.7%). Regarding participants’ level of education, comparable frequencies were seen among the participants, about 62 (28%) were in the fourth year, 56 (25%) in the fifth year, 49 (22%) in the sixth year, and 56 (25%) were dental interns. Table 1 presents the demographic data of the participants in the study.

**Table 1:** Demographic characteristics of the survey respondents.

Characteristics	Number (n)	Percentage (%)
Gender		
Male	99	44.40%
Female	124	55.60%
Total	223	100%
University		
Taibah University	113	50.70%
Umm Al-Qura University	76	34.10%
Qassim University	34	15.20%
Education level		
Fourth year	62	27.8%
Fifth year	56	25.1%
Sixth year	49	22%
Intern	56	25.1%

Table 2 shows 15 questions divided into three sections, i.e., knowledge, attitude, and practice, while surveying the students, along with the number and percentage of participants and their responses. The results show that around 107 (48%), 180 (80.7%), and 45 (20.2%) participants adhered to the best or correct answers for each section, respectively.

**Table 2:** Knowledge, attitude, and practice questions and the number and percentage distribution of students and interns.

Question	Answers	Number (n) and percentage (%)
What is the safest period for treating pregnant women?	First trimester	6 (2.7%)
	Second trimester	199 (89.2%)
	Third trimester	18 (8.1%)
What is the most common oral finding or disease experienced by pregnant women?	Erosion	9 (4.0%)
	Dental caries	1 (0.4%)
	Periodontitis	63 (28.3%)
	Gingivitis	148 (66.4%)
	Ulcers	2 (0.9%)
Do you think periodontal disease during pregnancy can cause (or be associated with) low birth weight?	Yes	81 (36.3%)
	No	28 (12.6%)
	I don’t know	114 (51.1%)
Are you familiar with the FDA’s classification system for medications?	Yes	105 (47.1%)
	No	118 (52.9%)
What is the most comfortable seating position for pregnant women during dental treatment?	The patient should lie on their left side with their right buttocks and hip slightly raised by 15 degrees	141 (63.2%)
	The head and heart should be parallel to the floor and the feet should be elevated slightly	12 (5.4%)
	Upright position	59 (26.5%)
	Supine position	11 (4.9%)
Are pregnant women at a high risk of developing dental caries?	Agree	162 (72.6%)
	Disagree	34 (15.2%)
	I don’t know	27 (12.1%)
Is taking dental X-rays safe during pregnancy?	Agree	166 (52.0%)
	Disagree	103 (46.2%)
	I don’t know	4 (1.8%)
What is the safest local anesthesia to use in pregnant patients?	Lidocaine	183 (82.1%)
	Articaine	14 (6.3%)
	Bupivacaine	16 (7.2%)
	Contraindicated	10 (4.5%)
Was the dental management information provided during undergraduate training for pregnant women sufficient?	Yes	112 (50.2%)
	No	44 (19.7%)
	Not sure	67 (30.0%)
What is your preferred source of information regarding the dental management of pregnant women?	Brochure	2 (0.90%)
	Lectures and books	173 (77.6%)
	Internet	34 (15.2%)
	Video	14 (6.3%)
Which antibiotic would you prefer to prescribe to pregnant women?	Amoxicillin	162 (72.6%)
	Tetracycline	7 (3.1%)
	Erythromycin (JA1)	11 (4.9%)
	Contraindicated	43 (19.3%)
When it comes to using analgesics for pregnant women, which one would you prefer to prescribe?	Diclofenac	6 (2.7%)
	Paracetamol	193 (86.5%)
	Ibuprofen	10 (4.5%)
	Celecoxib	3 (1.3%)
	Contraindicated	11 (4.9%)
Do you feel confident treating pregnant women?	Yes	85 (38.1%)
	No	138 (61.9%)
What actions should be taken if a pregnant woman in the third trimester experiences supine hypotension while sitting in a dental chair?	Sit the patient in an upright position	35 (13.3%)
	Roll the patient onto their left side	141 (62.9%)
	Elevate the patient’s legs	47 (23.8%)
When does active dental infection in pregnant women require management?	Immediately	129 (57.8%)
	Postponed treatment until delivery	21 (9.4%)
	Prescribe medication for pain relief until delivery	33 (14.8%)
	Referred to a gynecologist for his opinion	40 (17.9%)

Using the chi-square test at a significance level of p < 0.05), the breakdown of participants’ knowledge scores against their gender revealed no statistical significance (p = 0.034). At the same time, a statistically significant difference was found between the participants’ knowledge and their level of education (p < 0.001). Approximately 107 (48%) participants showed good knowledge, with dental interns being the vast majority (163, 73.2%), as shown in Table 3.

**Table 3:** Assessment of the level of knowledge for gender and education level.

Level of participants’ knowledge				
Variable	Poor, n (%)	Adequate, n (%)	Good, n (%)	P-value
Gender				
Male	3 (3.0%)	43 (43.4%)	53 (53.5%)	0.034
Female	0 (0.0%)	70 (56.5%)	54 (43.5%)	
Education level				
Fourth year	0 (0.0%)	40 (64.5%)	22 (35.5%)	<0.001
Fifth year	1 (1.8%)	23 (41.1%)	32 (57.1%)	
Sixth year	2 (4.1%)	35 (71.4%)	12 (24.5%)	
Intern	0 (0.0%)	15 (26.8%)	41 (73.2%)	
Average	1.5%	51%	47.5%	

Table 4 illustrates the assessment of the participant’s level of attitude concerning their gender and educational level. The results revealed that 181 (81.2%) participants had a good attitude toward the dental considerations of pregnant women. In contrast, no statistically significant associations were found between their gender or educational level, with p-values of 0.246 and 0.119, respectively.

**Table 4:** Assessment of the level of attitude for gender and education level.

Level of participants’ attitude				
Variable	Poor, n (%)	Adequate, n (%)	Good, n (%)	P-value
Gender				
Male	3 (3.0%)	15 (15.2%)	81 (81.8%)	0.246
Female	10 (8.1%)	15 (12.1%)	99 (79.8%)	
Education level				
Fourth year	6 (9.7%)	14 (22.6%)	42 (67.7%)	0.119
Fifth year	2 (3.6%)	7 (12.5%)	47 (83.9%)	
Sixth year	3 (6.1%)	4 (8.2%)	42 (85.7%)	
Intern	2 (3.6%)	5 (8.9%)	49 (87.5%)	
Average	5.75%	13.05%	81.2%	

Analysis of the participant’s level of practice, as shown in Table 5, illustrates that 101 (45.25%) participants possessed poor practice levels. A significant association between their educational level and level of practice was found (p < 0.001), while no statistical significance was found concerning their gender (p = 0.194).

**Table 5:** Assessment of the level of practice for gender and education level.

Level of participants’ practice				
Variable	Poor, n (%)	Adequate, n (%)	Good, n (%)	P-value
Gender				
Male	40 (40.4%)	40 (40.4%)	19 (19.2%)	0.194
Female	62 (50.0%)	36 (29.0%)	26 (21.0%)	
Education level				
Fourth year	39 (62.9%)	18 (29.0%)	5 (8.1%)	<0.001
Fifth year	26 (46.4%)	23 (41.1%)	7 (12.5%)	
Sixth year	22 (44.9%)	19 (38.8%)	8 (16.3%)	
Intern	15 (26.8%)	16 (28.6%)	25 (44.6%)	
Average	45.25%	34.37%	20.38%	

## Discussion

Numerous international and local studies have been performed to evaluate dental interns’ understanding of the dental management of pregnant women [1,15,16]. However, the variety of dental schools and the wide geographic area of Saudi Arabia require further research to cover more dental schools from other areas. This study aimed to assess dentistry students’ and interns’ knowledge and attitude and their confidence level and preparedness to practice safe dental care for expectant mothers in three dental schools in Saudi Arabia, namely, the dental schools at Taibah University, Umm Al-Qura University, and Qassim University.

The collective response rate of the participants was considered to be very high (97%), which allowed a proper analysis of the sample. However, the distribution of participants according to their university status would raise some concerns initially, as approximately half of the participants were from Taibah University. The total number of enrolled dental students in each dental school can justify this finding. The average number of students in the class is reported to be around 30, 25, and 18 dental students at the dental schools of Taibah University, Umm Al-Qura, and Qassim University, respectively. Considering these numbers while interpreting the results showed a relatively fair distribution among the participation rate from each university. Moreover, the frequencies of the participants according to their academic level showed a cohesive range of participation among the sample.

Regarding the participants’ knowledge, the survey examined this domain with eight questions. The participants exhibited an adequate level of understanding of the basic information regarding dental considerations while managing pregnant women. For example, 199 (89.2%) participants recognized that the second trimester of pregnancy is the safest period to perform dental procedures. This finding is consistent with prior reports, as 87% of dental interns at a private dental school in Saudi Arabia [1] and 83% of several governmental dental schools [8] agreed that pregnant women can safely undergo dental work during the second trimester. Another study among dental interns in India reported similar results as well [15]. Awareness of dental practitioners regarding the best time to provide dental care is essential to avoid unnecessary complications. According to the recommendations of the American Dental Association, between the 14th and 20th gestational weeks is the ideal time to schedule elective dental care for pregnant women.

The question about the relationship between periodontal disease during pregnancy and low birth weight revealed a contradicting finding to what has been reported earlier. In this study, only 36.3% of the participants knew about the relationship between periodontal problems and low birth weight. This rate is considerably lower than what had been reported by Jayabalan and Muthusekhar, where 87% of their sample knew about that relationship [16]. Another knowledge level was reported by Tantradi and Madanshetty, as 59% of the interns in their sample showed knowledge about this association [15]. This observation is alarming as it exposes the limited knowledge of dental students and new graduates regarding the impact of periodontal diseases on healthy pregnancy. Periodontal disease during pregnancy has been associated with several negative complications during pregnancy, including preeclampsia, low birth weight, and preterm birth [17]. Therefore, this should be further emphasized during the theoretical and clinical training of dental students and interns.

The majority of the participants in this study chose the correct or best answer regarding antibiotics, local anesthetics, and pain medications with pregnant women. However, almost 118 (52.9%) needed to familiarize themselves with the FDA’s medication classification system [18]. This finding could be attributed to the learning methodology of the students, as they knew the proper medications from lectures without focusing on the source of the information in the lecture. A similar result was reported by Swapna et al., as 50% of the dental interns reported little knowledge of the FDA’s classification [1]. The need for knowledge about the FDA’s classification was also reported with a higher rate (80%) in a dental intern sample in India [15].

Regarding the utilization of dental radiographs with pregnant women, 103 (46.2%) study participants considered such a procedure unsafe. Two previous studies showed that 36% [1] and 27% [8] of the interns from different regions in Saudi Arabia favored not taking dental radiographs. A higher rate was reported in an Indian sample where 88% of the respondents considered it to be an unsafe procedure [15]. The differences in response rate could be due to the type of subjects, as our sample included dental students from different levels in addition to interns. These numbers, however, reflect the need to improve the knowledge of dental students and interns regarding the safety of dental X-ray utilization for pregnant women. It is well-established that exposure to dental ionizing radiation is safe at all stages of pregnancy and has no potential impacts on in-utero birth defects [19]. Therefore, dental professionals should remain educated regarding the safety of dental radiograph exposure to provide better oral healthcare to pregnant women. It is important to educate students and interns about the safety of dental X-rays for pregnant women. The amount of radiation used in dental X-rays is very low and safe for pregnant women [20]. However, it is necessary to follow the guidelines and use proper safety equipment while using X-rays to ensure the safety of both the patient and the fetus. It is crucial to inform individuals about the potential hazards and risks associated with radiation exposure.

The attitude section investigated the student’s responses through four close-ended questions. When students were asked about the sufficiency of the provided information in the curriculum regarding the dental management of pregnant women, 112 (50%) participants found it sufficient. This finding could be affected in our study sample as the students were included from different levels of education. However, there was no statistical association between the attitude level and the level of education in the sample. Tantradi and Madanshetty also reported a similar result, as 48% of their sample confirmed receiving theoretical information [15]. Moreover, 39% was reported in another study [21]. Nevertheless, when the same question was asked to the dental interns of a multicenter Saudi dental schools study, they responded with lower satisfaction with the provided information within the reported programs, as almost 30% of dental interns found the curriculum information about dental management of pregnant women to be enough [8]. Another study reported an even lower rate of knowledge acquisition through the curriculum by a sample of dental interns in Karachi, which was reported to be around 24% [22]. These results would indicate that dealing with pregnant women is not given proper attention within the dental undergraduate curricula.

Concerning the primary preferred source of knowledge, 173 (77.6%) undergraduate students preferred lectures and textbooks over pamphlets, the Internet, and videos. This attitude ensures that students are equipped with evidence-based information. However, this infers that students likely depend on lectures and the teacher’s ability to convey the necessary information. Many published studies have shown that students prefer lectures and textbooks but with lower dependency rates, ranging between 33% and 61% [1,8,10,21].

The practice part of the survey consisted of three questions. When the sample was asked about their confidence level to treat expectant mothers, about 138 (61.9%) expressed their hesitation to manage such cases. This result confirms what has been previously published as many studies have demonstrated variable intern confidence levels that infer questionable practice and require corrective actions [1,15,16].

It is the responsibility of the dentist to promptly address dental caries causing acute infection and pain in a pregnant woman, regardless of the stage of pregnancy [23,24]. This is because leaving an untreated active infection can pose a greater risk than the potential risk associated with performing the necessary treatment. Additionally, febrile illness and sepsis can increase the likelihood of miscarriage [25]. The present study showed that over half of the participants would provide immediate treatment for acute dentoalveolar infection. This abides by the evidence-based clinical recommendation in which necessary procedures can be provided at any stage of pregnancy [26]. In comparison with the findings of another study, it was reported that only 23% of the participants would proceed with prompt treatment [1]. Furthermore, another study revealed that 8.6% of the participants were willing to provide immediate treatment [16].

It is worth noting that the present study sample included different levels of education, which might make the comparison and discussion of the results noncohesive, as most of the reports included dental interns solely.

Limitations of the study included a limited sample size and a focus on students and interns in only three universities. Hence, increasing the number of participants and universities could make the conclusion valid to the country of Saudi Arabia, not just a specific area. Furthermore, the methodology showed a gap between the different levels of education. Therefore, further studies, including students, are recommended to explore the effectiveness of the courses provided within the curriculum concerning the dental management of pregnant women.

## Conclusions

The majority of dental students and interns in this study (61.9%) showed a lack of confidence in managing pregnant patients. They exhibited adequate knowledge in certain aspects and positive attitudes regarding dental considerations in pregnant women. However, they presented low awareness regarding the association between periodontal disease and negative pregnancy complications, including low birth weight and preterm birth. Therefore, further training during their undergraduate studies, as well as continuous education after graduation, are deemed necessary to enhance their proficiency in treating pregnant patients. This enhancement is recommended through incorporating exposure to pregnant women within the undergraduate clinical training to raise the confidence level of future Saudi dentists while providing dental care for this unique group of patients after graduation.

## Appendices

Survey used in the study

Demographic data

What is your gender?

A. Male

B. Female

What is your university?

A. Umm Al-Qura University

B. Taibah University

C. Qassim University

What is your educational level?

A. Third-year dental student

B. Fourth-year dental student

C. Fifth-year dental student

D. Sixth-year dental student

E. Intern

Section one: self-assessment

Was the dental management information provided during undergraduate training for pregnant women sufficient?

A. Yes

B. No

C. Not sure

What is your preferred source of information regarding the dental management of pregnant women?

A. Brochure

B. Lectures and books

C. Internet

D. Video

Which antibiotic would you prefer to prescribe to pregnant women?

A. Amoxicillin

B. Tetracycline

C. Erythromycin

D. Contraindicated

When it comes to using analgesics for pregnant women, which one would you prefer to prescribe?

A. Diclofenac

B. Paracetamol

C. Ibuprofen

D. Celecoxib

E. Contraindicated

Section two: self-knowledge

What is the safest period for treating pregnant women?

A. First trimester

B. Second trimester

C. Third trimester

What is the most common oral finding or disease experienced by pregnant women?

A. Erosion

B. Dental caries

C. Periodontitis

D. Gingivitis

E. Ulcers

Do you think periodontal disease during pregnancy can cause (or be associated with) low birth weight?

A. Yes

B. No

C. I don’t know

Are you familiar with the FDA’s classification system for medications?

A. Yes

B. No

What is the most comfortable seating position for pregnant women during dental treatment?

A. The patient should lie on their left side with their right buttocks and hip slightly raised by 15 degrees

B. Head and heart parallel to the floor and feet elevated slightly

C. Upright position

D. Supine position

Are pregnant women at a high risk of developing dental caries?

A. Agree

B. Disagree

C. I don’t know

Is taking dental X-rays safe during pregnancy?

A. Agree

B. Disagree

C. I don’t know

What is the safest local anesthesia to use in pregnant patients?

A. Lidocaine

B. Articaine

C. Bupivacaine

D. Contraindicated

Section three: practice

Do you feel confident treating pregnant women?

A. Yes

B. No

What actions should be taken if a pregnant woman in the third trimester experiences supine hypotension while sitting in a dental chair?

A. Sit the patient in an upright position.

B. Roll the patient onto their left side

C. Elevate the patient’s legs.

When does active dental infection in pregnant women require management?

A. Immediately

B. Postponed treatment until delivery

C. Prescribe medication for pain relief until delivery

D. Referred to a gynecologist for his opinion
